# Identifying Key Regulators in Odorant Receptor Trafficking

**DOI:** 10.1523/JNEUROSCI.0454-25.2025

**Published:** 2025-10-22

**Authors:** Hsiu-Yi Lu, Hiroaki Matsunami

**Affiliations:** ^1^Departments of Molecular Genetics and Microbiology, Duke University School of Medicine, Durham, North Carolina 27710; ^2^Neurobiology, Duke University School of Medicine, Durham, North Carolina 27710

**Keywords:** olfactory receptor, chemical senses, protein trafficking, transcriptomics, GPCR

## Abstract

Odor detection in mammals is primarily mediated by odorant receptors (ORs), the largest family of G-protein-coupled receptors, expressed in olfactory sensory neurons (OSNs; Buck and Axel, 1991). However, most ORs exhibit little or no cell surface expression in nonolfactory cell types (Lu et al., 2003; Hague et al., 2004). While the accessory protein RTP1 and RTP2 enhance the expression of certain ORs, we hypothesized that additional proteins coregulated with RTP1 and RTP2 during OSN maturation may further enhance OR cell surface expression (Saito et al., 2004; Zhuang and Matsunami, 2007). To test this, we developed a computational pipeline based on publicly available single-cell transcriptomic data to create an interactive tool for exploring gene expression during OSN maturation. Using this tool, we identified genes coregulated with Rtp1 during olfactory development. Three genes—Gfy, Clgn, and Syt1—exhibited transcriptional profiles similar to Rtp1. When coexpressed with an OR, these genes, in combination with Rtp1, promoted cell-surface expression of the tested ORs. Importantly, we demonstrated a physical interaction between Syt1 and an OR (Or1ad1) via co-immunoprecipitation, suggesting a novel role for Syt1 in OR trafficking or stabilization. We also observed localization of Syt1 protein in OSN cilia in both male and female mice. Together, these findings provide new insights into OSN development and the molecular mechanisms underlying OR biogenesis, paving the way for further research into the functional regulation of the olfactory systems.

## Significance Statement

Odorant receptors (ORs) are notoriously difficult to express in heterologous systems, hindering the study of their structure and function. In this study, we identify accessory proteins, including Synaptotagmin 1 (Syt1), a protein best known for its role in regulating synaptic neurotransmitter release, which enhances OR cell-surface expression. We identify coregulated genes such as Gfy, Clgn, and Syt1, shedding light on olfactory sensory neuron (OSN) development and OR biogenesis. These findings have broader implications for understanding neuronal development and G-protein-coupled receptor regulation. Additionally, we developed an interactive tool for exploring gene expression during OSN maturation, providing a resource for the scientific community. By addressing the challenges of OR biogenesis and introducing new tools, this study advances molecular biology, neuroscience, and biotechnology.

## Introduction

Olfaction relies on the function of olfactory sensory neurons (OSNs) located in the olfactory epithelium (Buck and Axel, 1991). The vast majority of OSNs express odorant receptors (ORs), which constitute the largest family of G-protein-coupled receptors (GPCRs) and are responsible for detecting a wide array of odorants (Spehr and Munger, 2009; DeMaria and Ngai, 2010). The human genome encodes ∼400 intact OR genes, while mice possess over 1,000, reflecting the complexity and diversity of the olfactory system (Niimura et al., 2014). The proper development and OSNs is critical for olfactory function. OSNs undergo a well-defined maturation process, transitioning from globose basal cells (GBCs) to immediate neural precursor (INP), immature OSNs (iOSNs), and eventually to mature OSNs (mOSNs; DeMaria and Ngai, 2010). During this process, each neuron chooses and expresses a specific OR type. This choice, along with the cell surface expression of the ORs, is tightly regulated and essential for maturation (Malnic et al., 1999; DeMaria and Ngai, 2010; McClintock, 2015; Dalton, 2017; Sharma et al., 2017; Shayya et al., 2022).

Early in OSN development, the unfolded protein response (UPR) pathway is triggered by the accumulation of ORs in the endoplasmic reticulum (ER), followed by the release of UPR response. This transient UPR activity is necessary for proper OR gene choice and is coupled with dynamic gene expression during OSN maturation to ensure proper OR folding and trafficking. This dynamic gene expression process is crucial for mOSN differentiation and functionality. However, current tools for visualizing gene expression changes are often limited to cell types or clusters, rather than providing insights into continuous developmental trajectories. Thus, to understand OSN maturation and the regulatory networks important for OR folding and cell surface expression, it is crucial to investigate the gene expression changes during this critical window.

The ability to express ORs on the cell surface in nonolfactory cells is pivotal for identification of OR–ligand interactions using cell-based assays and for advancing our understanding of OR biogenesis. However, achieving OR surface in heterologous systems has historically been challenging (Lu et al., 2003; Hague et al., 2004). Misfolding and poor trafficking often prevents ORs from reaching the cell membrane, which significantly hampers OR–ligand interaction studies. The discovery of receptor-transporting proteins, RTP1 and RTP2, improved the surface expression of many ORs in heterologous systems (Saito et al., 2004; Zhuang and Matsunami, 2007). Yet, even with RTP1 and RTP2, not all ORs achieve sufficient surface expression, indicating a need for additional chaperones or regulatory mechanisms.

Here, we focused on identifying coregulated chaperones with Rtp1during OSN maturation. By leveraging the coexpression patterns of Rtp1, we aimed to uncover additional proteins that work synergistically, overcoming the limitations of previous approaches and enhancing OR surface expression in heterologous systems. This strategy builds on the regulatory gene expression network during OSN development, providing insights into molecular chaperones critical for OR functionality and addressing gaps in heterologous system.

## Materials and Methods

### Data usage

Mouse whole olfactory mucosa Drop-seq single-cell RNA-seq data and metadata were downloaded from GSE151346 (Brann et al., 2020) and GSE173947 (Tsukahara et al., 2021). Additional bulk-RNA-seq of FACS-sorted OSNs are downloaded from GSE198886 (Shayya et al., 2022). Mouse sensory cilia Proteome were downloaded and reanalyzed from supplemental materials (Kuhlmann et al., 2014).

### Processing and reanalysis of WOM single-cell RNA-seq

All downstream analysis is processed in Python, using anndata (Virshup et al., 2023) and scanpy (Wolf et al., 2018) for preprocessing. Downloaded gene expression matrix and Metadata were indexed and merged into the anndata object by matching barcode indices and filtering out cells absent in the metadata.

### Data preprocessing and quality control

Normalization was performed. Counts per cell are each normalized to a total of 10,000 counts per cell to correct for differences in sequencing depth across samples. Anndata object is followed by a logarithmic transformation to stabilize variance across the dataset. Standard scanpy single-cell preprocessing steps are followed. Cells exhibiting high mitochondrial gene content were removed to eliminate potentially compromised cells. Additionally, specific genes known to introduce noise or bias, such as mitochondrial and Malat genes, were excluded from the dataset to improve the quality of downstream analyses. Subsets of cells relevant to the study, such as OR genes, were specifically excluded from dimensionality reduction and clustering. Scanpy principal component analysis (PCA) was applied to reduce dimensionality of the gene expression data. Following PCA, neighborhood graph of the cells was constructed with n_neighbors = 10 and n_pcs = 40. Uniform manifold approximation and projection (UMAP) is implemented with the default scanpy parameters as resolution does not affect our downstream analysis and is purely used for visualization purpose (McInnes et al., 2018).

### Diffusion pseudotime and gene expression quantification across developmental time points

Diffusion pseudotime (DPT) was calculated to elucidate developmental trajectories within OSNs, with a more continuous precision in comparison with the discrete cluster-based cutoffs. The root cell was defined by globose basal cell due to its distinct expression profile. Individual gene expression levels were quantified across a continuum of developmental pseudotime bins by taking the average counts of all cells within the same DPT bin. Additionally, four distinct forms of the count data were utilized: normalized counts, logarithmized counts, normalized counts adjusted for the maximum expression of each gene, and logarithmized counts similarly adjusted. Root mean squared error (RMSE) was calculated for each gene to evaluate the variability of gene expression relative to a reference gene. This RMSE metric was used in ranking genes according to their relevance across different developmental timepoints. These rankings were subsequently used to discern the impact of various data preprocessing strategies, such as normalization versus logarithmic transformation, on gene expression patterns. The integration of results from all count forms at the conclusion of the study allowed for the identification of genes coexpressing with reference gene without bias toward any counts method. All final outputs, including detailed gene expression profiles, were integrated to online application for browsing.

### Construction and amplification of OR and gene of interest expression constructs

#### Primer design and preparation

Primers for cloning ORs or gene of interest into the pCI vector were designed to include MluI and NotI restriction sites for consistency, if Mlul and NotI sites are included in the target sequences other unique restriction sites are used instead. In general, the 5′ forward primer included an MluI linker sequence [AAACGCGT(ATG)], while the 3′ reverse primer included a NotI linker sequence (TTGCGGCCGC). Each primer had a unique sequence length of 18–22 nucleotides with an estimated melting temperature (Tm) of 56 or 58°C, accounting for 2°C per A/T and 4°C per G/C base pairing. Primers were reconstituted to 100 µM in deionized water (dW) and diluted to a working concentration of 5 µM.

#### DNA amplification

For DNA amplification using Phusion polymerase, reactions were set up as follows: 1 µl C57BL6 purified cDNA (0.2 ng/µl), 2 µl of 5× Phusion buffer (Thermo Fisher Scientific: F-549L), 0.1 µl of Phusion Hot Start II polymerase, 1 µl of 2 mM dNTP mix, and 1 µl of each forward/reverse primer at 5 µM. The volume was adjusted to 11.1 µl with water. Amplification conditions involved an initial denaturation at 98°C for 30 s, followed by 30 cycles of 98°C for 5 s, a touchdown from 65°C to 55°C decreasing 0.5°C per cycle for 15 s, and an extension at 72°C for 1 min per kb of target length. A final extension at 72°C for 5 min and a 10°C hold is applied. Following amplification, 1 µl of PCR product can be used to visualize product amplification on a 1% agarose gel.

#### DNA purification

Following amplification, the remaining PCR product was purified using the Qiagen PB and PE buffer system from QIAquick PCR Purification Kit (Qiagen: catalog #28104). The PCR product was first bound to QIAquick spin columns in 200 µl of PB buffer, washed with 750 µl of PE buffer, dried with 2 min spin, and eluted in 10 µl of elution buffer (EB).

#### Restriction enzyme digestion

For vector digestion, a mixture was prepared containing 9 µl of purified DNA product, 2 µl of NEBuffer3 (B7003S), 0.5 µl each of MluI (R0198) and NotI (R0189), 0.2 µl BSA(B9000) and 8 µl of water per sample. Samples are incubated at 37°C for 2 h. Following digestion, digested products are combined with 6× dye (R0611) and loaded into 2% agarose gel for 30 min of gel electrophoresis. Minimize and use short wave UV to visualize and cut band of expected product size directly into 450 µl QG buffer from QIAquick Gel Extraction Kit (catalog #28704) in an Eppendorf tube. Gel is dissolved by incubating tube in 50–65°C chamber for 10 min while mixing occasionally. Dissolved products are then passed through QIAquick spin column and purified with 150 µl isopropanol, 500 µl QG buffer, 750 µl PE buffer, dried for 2 min, and eluted with 10 µl of EB.

#### Ligation and transformation

Ligation was performed by combining 3 µl of insert DNA, 0.5 µl of Rho-pCI vector, 0.5 µl of NEB T4 DNA ligase (M0202), 0.5 µl of NEB ligation buffer (B0202), and 0.5 µl of water. The ligation mixture was incubated at room temperature for over 1 h or overnight at 4°C. Competent cells were then transformed with 2.5 µl of the ligation product, kept on ice for 10 min, heat-shocked if necessary for 30 s at 37–42°C, and plated on LB-amp plates for overnight incubation at 37°C.

#### Colony screening

Colony PCR was utilized to confirm insert presence using forward (pCI5′A: CTCCACAGGTGTCCACTC) and reverse (pCI3′A: CACTGCATTCTAGTTGTGG) pCI primers. Colonies are picked and diluted in 20 µl of water. For colony PCR NEB Taq DNA polymerase is used (M0273), and a mixture of 2 µl diluted colony, 1 µl 10× buffer, 1 µl of 2 mM dNTP, 1 µl of pCI5ʹ primer (5 µM), 1 µl of pCI3ʹ primer (5 µM), and 5 µl of Taq DNA Polymerase is used for PCR amplification. Amplification conditions involved an initial denaturation at 95°C for 15 min, followed by 25 cycles of 95°C for 15 s, 55°C for 15 s, and an extension at 72°C for 1 min per kb of target length. A final extension at 72°C for 1 min and a 4°C hold is applied. Following amplification 1 µl of PCR product can be used to visualize product amplification on a 1% agarose gel.

#### Extraction of purified plasmid

Successful clones were cultured in 5 ml of 2XYT-amp (100 µg/ml) at 37°C for 18–24 h. Plasmids are then extracted and purified from bacterial culture using ZymoPURE Plasmid Miniprep Kit (catalog #D4212) following manufacturer’s protocols. In short, colonies were harvested by centrifugation. The cell pellet was resuspended in resuspension buffer, lysed using the lysis buffer, and neutralized in neutralization buffer and cellular debris, and SDS was removed by centrifugation. The supernatant containing the plasmid DNA was filtered and transferred onto the Zymo-Spin Column for purification. The plasmid DNA was washed to eliminate impurities and subsequently eluted TE buffer. Plasmids are all diluted to 100 ng/µl and stored at 4°C.

### Quantifying cell surface expression of olfactory receptors

HEK293T cells were cultured under standard conditions in Minimal Essential Medium (MEM) supplemented with Earle's salts, ʟ-glutamine, 10% fetal bovine serum, and 1% penicillin-streptomycin. Cultivation took place in a humidified incubator at 37°C and 5% CO_2_. For the expression studies, OR genes were cloned into the Rho-pCI mammalian expression vector, with an epitope Rho-tag, comprising the first 20 amino acids of human rhodopsin, attached to the N-terminal end of the OR sequence. Cells were seeded in 35 mm 6-well plates at ∼2 × 10^5^ cells per well (20% confluency) overnight.

After 18–24 h, transient transfection of HEK293T cells was performed using Lipofectamine 2000, with DNA plasmid concentrations not exceeding 2 µg per well. DNA plasmids transfected includes GFP (100 ng), short receptor-transporting protein 1 (RTP1S, 100 ng; Saito et al., 2004), OR (500–1,000 ng), and additional genes of interest cloned in pCI. Following a further 18–24 h incubation to permit gene expression, cells were detached using a mixture of 1 ml of Cell Stripper (Corning) and 1 ml of ice-cold PBS mixture (phosphate-buffered saline, PBS Invitrogen, containing 2% FBS and 15 mM sodium azide, NaN3). The detached cells were transferred into 5 ml round-bottom polystyrene tubes (Falcon) and maintained on ice. Cells were centrifuged at 4°C, resuspended in the PBS mixture, and prepared for staining.

Surface expression of ORs was assessed using a double antibody staining method. Initially, cells were stained with 50 µl at 1/400 (v/v) dilution of the primary mouse monoclonal anti-Rhodopsin 4D2 (Rho4D2) antibody diluted in PBS mixture for 30 min on ice. After washing with the 2 ml PBS mixture and spun down, cells were stained again with 50 µl of 1/100 (v/v) dilution of phycoerythrin (PE)-conjugated anti-mouse IgG secondary antibody (Jackson Immunologicals) diluted in PBS mixture for another 30 min on ice in the dark. Subsequent washes were performed before adding 500 µl of 1/500 (v/v) of 7-amino-actinomycin D (Calbiochem: 129935) diluted in PBS mixture to label dead cells, and cells were prepared for immediate flow cytometric analysis.

Flow cytometry was conducted using a BD FACScanto flow cytometer, with gating controls set to identify spherical, single, viable, and GFP-positive cells. PE fluorescence intensities were recorded for 10,000 gated cells per sample. The geometric mean of PE fluorescence, calculated using FlowJo v10.8.1, served as a proxy for the level of cell surface expression. An empty Rho-pCI plasmid served as a negative control to establish the baseline level of PE expression per experiment.

### Olfactory receptor co-immunoprecipitation (co-IP)

HEK293T cells were plated in 100 mm dishes at ∼25% confluency. On the following day, cells were transfected using Lipofectamine 2000, following the same protocol as FACS transfections but scaled up sixfold from 6-well conditions. Transfection mixtures were further diluted to 10 ml total volume per 100 mm dish. For the OR + RGC + Syt1 condition, plasmids encoding Rho-tagged Or1ad1, Rtp1 s, Gfy, Clgn, and Syt1 were cotransfected. Control conditions included either Rho-pCI + RGC + Syt1 (no OR) or OR + RGC (no Syt1).

Cells were washed with ice-cold PBS and lysed with 1.2 ml ice-cold lysis buffer (50 mM Tris-HCl, 150 mM NaCl, 1% NP-40) containing protease inhibitor mix (Complete, Mini; Roche). Lysates were incubated for 2 h at 4°C with gentle rotation. After centrifugation at 14,000 × *g* for 15 min at 4°C, the supernatant was collected. A portion of each lysate (30–50 µl) was saved as the input sample for western blot. Input samples were mixed with 4× SDS buffer and incubated with 1 M DTT (final 250 mM) for 1–2 h at room temperature.

To immunoprecipitate the OR, lysates are first precleared with Protein A/G magnetic beads (Pierce 88802) washed with wash buffer (50 mM Tris-HCl, 150 mM NaCl, 0.5% NP-40) containing protease inhibitor mix. Lysates were then incubated with anti-Rho antibody (Rho4D2), and 1 µg antibody further diluted typically 1:1,000 dilution with lysate samples. Antibody binding was done overnight at 4°C with gentle agitation. Next, Protein A/G magnetic beads (Pierce 88802) were precleared by washing twice with wash buffer. A total of 25 µl beads (∼0.25 mg) were used per 500 µl lysate. Antibody-bound lysates were incubated with the beads for 1–2 h at room temperature with gentle mixing. Beads were then washed five times with 500 µl cold wash buffer for 3 min each, with gentle shaking, and collected using a magnetic rack. Pulldown samples were eluted from beads by incubating with 40–80 µl of 1.2× SDS buffer (no DTT) for 2 h at room temperature. After removal from beads, DTT was added and incubated for 1–2 h. All samples were then resolved by SDS-PAGE and analyzed by Western blot using Anti-Syt1 (ANR003) and anti-Rho antibodies.

### SDS-PAGE electrophoresis and Western blot

Samples were resolved on commercially available 4–20% Tris-Glycine gradient gels (Bio-Rad #4568094) using freshly prepared 1× running buffer (25 mM Tris base, 192 mM glycine, and 3.5 mM SDS). Prestained protein ladder (Thermo Fisher Scientific #26619, 5–10 µl) and 10–30 µl of each sample were loaded per lane. Electrophoresis was performed at 100 V for ∼60 min or until the dye front reached the bottom.

Proteins were transferred using wet transfer onto nitrocellulose membranes in chilled transfer buffer (48 mM Tris base, 39 mM glycine, and 20% methanol) using a Bio-Rad wet transfer system. Gels were pre-equilibrated in transfer buffer, and the transfer sandwich was assembled. The assembly was placed in the chamber, and the tank was filled with cold transfer buffer with a prefrozen cooling unit inserted. Transfer was carried out overnight at 90 mA (∼17 V) in a 4°C cold room. For total protein visualization, TGX Stain-Free gels were UV-activated for 1 min (Bio-Rad imager).

Membranes were blocked in 5% (w/v) nonfat dry milk in 1× TBS-T (50 mM Tris, 150 mM NaCl, and 0.5% Tween-20) for at least 1 h at room temperature. Primary antibody incubations were performed in sealed parafilm pouches using 4 ml of antibody solution prepared in 5% milk. Incubations were conducted for 1 h at room temperature or overnight at 4°C with shaking. The following primary antibodies were used: mouse anti-Rho 4D2 (1:1,000) and rabbit anti-Syt1 (1:1,000). Membranes were rinsed twice and washed three times in TBS-T for 10 min each. Secondary HRP-conjugated antibodies were diluted 1:4,000 in 5% milk and incubated for 60 min at room temperature. Membranes were again rinsed and washed three times in TBS-T for 10 min each. Protein bands were visualized using SuperSignal West Femto (#34094; Thermo Fisher Scientific).

### Mice

C57BL/6J mice (*Mus musculus*) were sourced from the Jackson Laboratory and housed under controlled conditions in the Matsunami laboratory at Duke University. The mice utilized in these experiments were 21 d postnatal male and female mice to ensure sufficient maturation of the olfactory organ. All animal husbandry practices and experimental procedures were conducted in accordance with the guidelines set by Duke University’s Institutional Animal Care and Use Committee (IACUC) and conducted in accordance with federal guidelines for the ethical treatment of laboratory animals.

### Tissue preparation for cryosectioning

Mice 21 postnatal age were killed using carbon dioxide inhalation followed by cervical dislocation to ensure death. Post-killing, the head was separated from the body, and dissections were performed to extract the olfactory epithelium for embedding. First, the lower jaw was detached to further expose the underlying structures. Initial cuts were made to remove the front teeth and the tip of the nose using scissors and the molars teeth located at the back of the jaw is also removed with tweezers individually. The olfactory epithelium structure was then detached by making careful incisions around its perimeter to detach it completely from the skull. The entire epithelium was immediately embedded in a 2 cm block of Optimal Cutting Temperature (O.C.T.) and frozen in liquid nitrogen. This prepared sample was then stored at −80°C until further processing for cryosectioning.

Olfactory epithelium tissue samples were sectioned using a cryostat to obtain 20-µm-thick slices. Sections were collected on Superfrost Plus slides and immediately stored at −80°C to preserve tissue integrity and RNA quality.

### Immunohistochemistry

The day of staining, slides were thawed and dried using a hair dryer before being loaded into a staining rack. Slides were then fixed in ice-cold 4% paraformaldehyde (PFA) for 15 min. For permeabilization, slides were treated with ice-cold methanol for 1 min and rinsed twice with PBS.

Following the wash, sections were blocked in blocking solution containing 5% skim milk (Nestle 12428935) in PBS to prevent nonspecific binding of antibodies. Primary antibodies [rabbit anti-ACIII, rabbit anti-Galpha, rabbit anti-syt1(ANR-003)], diluted in the same blocking solution, were then applied at a volume of 300 µl per slide in the blocking solution and incubated in a humidified chamber for either 1 h at room temperature.

After primary antibody incubation, slides were rinsed twice with PBS and washed three times for >5 min each at room temperature. Following wash, sections were incubated with fluorescently labeled secondary antibodies, (anti-rabbit Cy3 from Jackson ImmunoResearch) diluted 1:200 in blocking solution, for ∼45 min at room temperature in the dark.

After secondary antibody incubation, slides were rinsed twice with PBS and then stained with a 1/100,000 dilution of 1% Hoechst (bisBenzimide B2883) for 5 min at room temperature to visualize nuclei. Finally, sections were washed with PBS for 5 min at room temperature, rinsed with deionized water, and mounted using Mowiol mounting medium with glass coverslips. The slides are then allowed to dry and solidify before imaging, and slides can be stored at 4°C in the dark for a few weeks.

### Data analysis

Single-cell analyses were performed in Python 3.8 using Scanpy package (version 1.8.2). Custom scripts for data analysis and visualization are built using open-source Python libraries (pandas, numpy, matplotlib, plotly, sklearn, scipy, seaborn, and itertools). Scripts to replicate data analysis are available on GitHub (https://github.com/Justice-Lu/Chaperone_Analysis).

### Statistical analysis

Experimental data are presented as mean ± standard deviation (SD), based on results from at least three independent experiments. Statistical analysis was conducted in Python 3.11.13 using SciPy v1.16.0. Paired comparisons (e.g., samples with different condition measured in the same experiment) were analyzed using the paired Student’s *t* test (ttest_rel). For unpaired comparisons between two independent groups, the nonparametric Wilcoxon rank-sum test (ranksums) was applied. A table with all the statistical comparison values and description can be found at Table 1. A two-tailed *p* value <0.05 was considered statistically significant. Significance levels are denoted as follows: *p < 0.05, **p < 0.01, ***p < 0.001, and n.s. (not significant), *p* ≥ 0.05.

**Table 1.** Summary of statistical comparisons across experimental conditions

Figure	Group1	Group2	Test	Statistics	*p* value	*n*_Group1	*n*_Group2
Figure 3*B*	RhopCI	Or1ad1	ranksums	−3.69E+00	2.22 × 10^−4^	16	20
Figure 3*B*	Or1ad1	Or1ad1 + R	ranksums	−5.54E+00	3.022.22 × 10^−8^	20	22
Figure 3*B*	Or1ad1 + R	Or1ad1 + RGC	ranksums	−4.19E+00	2.832.22 × 10^−5^	22	18
Figure 3*B*	RhopCI	Or55b4	ranksums	−3.02E+00	2.562.22 × 10^−3^	12	11
Figure 3*B*	Or55b4	Or55b4 + R	ranksums	−4.06E+00	4.872.22 × 10^−5^	11	12
Figure 3*B*	Or55b4	Or55b4 + RGC	ranksums	−3.76E+00	1.692.22 × 10^−4^	11	9
Figure 3*B*	Or55b4 + R	Or55b4 + RGC	ranksums	−2.98E+00	2.842.22 × 10^−3^	12	9
Figure 3*C*	Or1ad1 + R	Or1ad1 + RGC	ttest_rel	−4.34E+00	1.172.22 × 10^−3^	12	12
Figure 4*A*	RhopCI	Or1ad1	ranksums	−2.63E+00	8.422.22 × 10^−3^	8	5
Figure 4*A*	RhopCI	Or1ad1 + Syt1	ranksums	−2.72E+00	6.582.22 × 10^−3^	8	4
Figure 4*A*	Or1ad1	Or1ad1 + R	ranksums	−2.93E+00	3.412.22 × 10^−3^	5	8
Figure 4*A*	Or1ad1 + R	Or1ad1 + R + Syt1	ranksums	−2.72E+00	6.582.22 × 10^−3^	8	4
Figure 4*A*	Or1ad1 + R + Syt1	Or1ad1 + RGC	ranksums	6.792.22 × 10^−1^	4.972.22 × 10^−1^	4	8
Figure 4*A*	Or1ad1 + R + Syt1	Or1ad1 + RGC + Syt1	ranksums	−2.04E+00	4.152.22 × 10^−2^	4	8
Figure 4*A*	Or1ad1 + RGC	Or1ad1 + RGC + Syt1	ranksums	−2.52E+00	1.172.22 × 10^−2^	8	8
Figure 4*A*	Or1ad1 + R	Or1ad1 + R + Syt1	ttest_rel	−8.10E+00	3.932.22 × 10^−3^	4	4
Figure 4*C*	Or1ad1	Or1ad1 + RGC	ranksums	−5.97E+00	2.372.22 × 10^−9^	22	27
Figure 4*C*	Or1ad1 + RGC	Or1ad1 + RGC + Syt1	ranksums	−4.7E+00	1.762.22 × 10^−6^	27	11
Figure 4*C*	Or1ad1 + RGC	Or1ad1 + RGC + Syt5	ranksums	−2.52E+00	1.172.22 × 10^−2^	27	6
Figure 4*C*	Or1ad1 + RGC + Syt1	Or1ad1 + RGC + Syt9	ranksums	3.12E+00	1.842.22 × 10^−3^	11	5
Figure 4*C*	Or1ad1 + RGC + Syt5	Or1ad1 + RGC + Syt9	ranksums	2.74E+00	6.172.22 × 10^−3^	6	5
Figure 4*D*	Or1ad1 + RGC	Or1ad1 + RGC + Syt1	ttest_rel	−4.15E+00	1.972.22 × 10^−3^	11	11
Figure 4*D*	Or1ad1 + RGC	Or1ad1 + RGC + Syt5	ttest_rel	−2.84E+00	3.622.22 × 10^−2^	6	6
Figure 4*D*	Or1ad1 + RGC	Or1ad1 + RGC + Syt1 + Syt5	ttest_rel	−4.69E+00	3.352.22 × 10^−3^	7	7

This table reports statistical comparisons between grouped conditions across multiple figures in the study. Each row corresponds to a specific comparison made in the indicated figure panel. Columns include the figure, comparison groups (Group1 and Group2), statistical test used (Wilcoxon rank-sum or paired *t* test), the test statistic value, the corresponding *p* value, and the corresponding number of samples.

## Results

### Gene expression trajectory in the neuronal lineage

**Figure 1.**
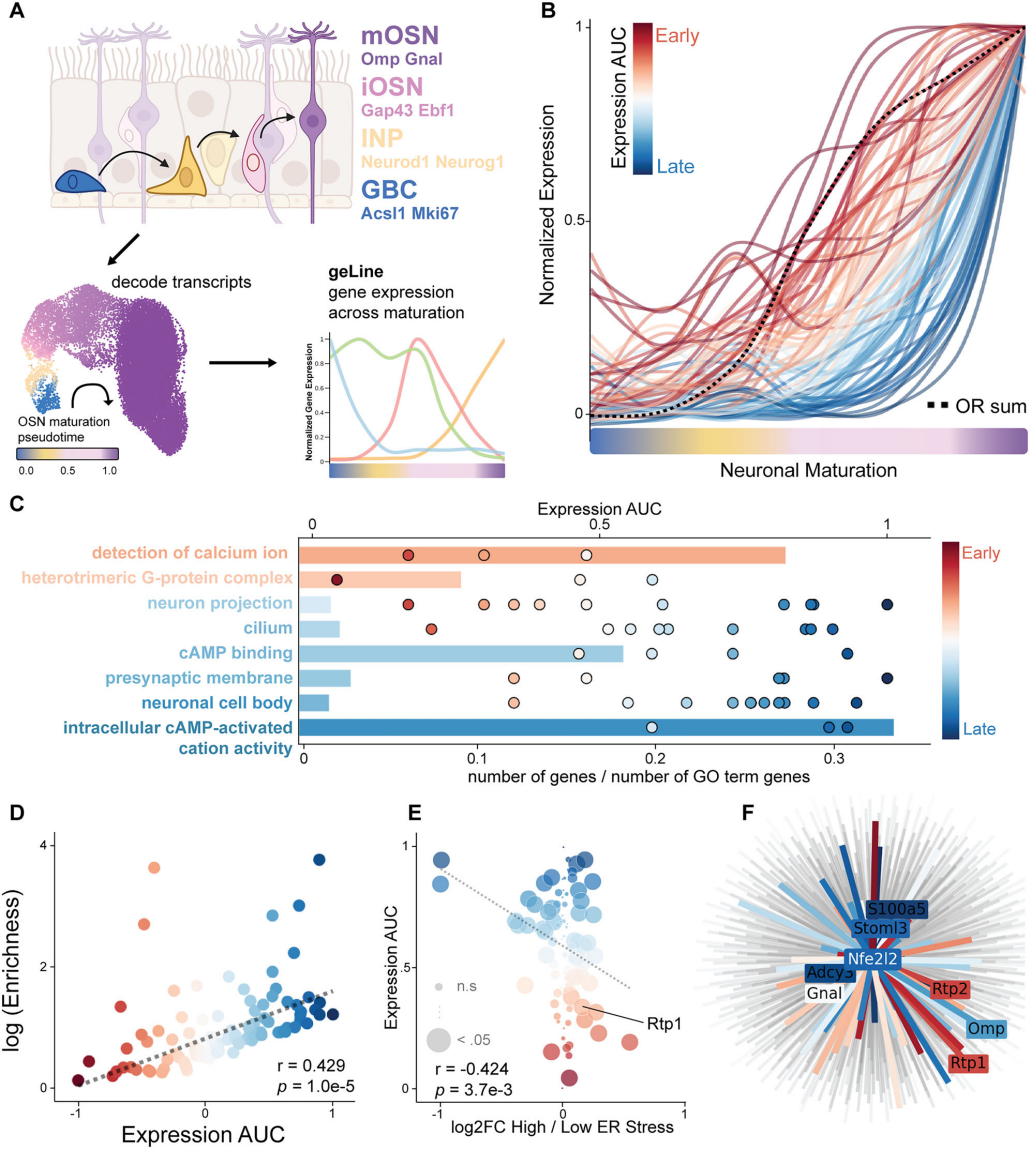
mOSN markers exhibit distinct functions with onset and trajectories during OSN maturation. ***A***, Schematic of the olfactory sensory neuron (OSN) differentiation lineage, annotated with key genetic markers. DPT derived from Scanpy analysis is used as a proxy for neuronal maturation and used to build geLine app. ***B***, geLine plot of the top 100 genes enriched in mature OSNs (mOSNs). Color denotes the area under the curve (AUC) for gene expression across the pseudotime trajectory, highlighting distinct temporal dynamics. ***C***, GO term enrichment analysis of the top mOSN-enriched genes. Bars represent the fraction of genes per GO term (bottom *x*-axis), with bar color indicating the average expression AUC (timing of GO term activation). Scatter dot colors denote the onset timing of individual genes (top *x*-axis). ***D***, Scatter plot showing the relationship between mOSN gene enrichment and normalized expression levels. ***E***, Correlation between expression onset (AUC) and differential gene expression between ER stressed OSNs, measured as log fold change (logFC). Pearson’s correlation coefficients are shown. Dot size corresponds to FDR (larger = FDR < 0.05), and dot color indicates expression AUC. ***F***, ARACNE-inferred transcriptional network of the ER stress-associated transcription factor Nfe2l2. Lines indicate regulatory interactions with downstream genes. Bold lines highlight genes among the top 100 mOSN-enriched genes. Node color reflects expression AUC.

Understanding gene expression and programming of cells during development requires more than just discrete groups of gene expression between different cell types. It is crucial to observe the transitions and trajectories of cells as they mature, since these transitions occur in a continuous space (Fig. 1*A*). By mapping these gene expression changes along the maturation continuum, we can gain deeper insights into the dynamic processes that govern neuronal development and differentiation. To effectively visualize gene expression trajectories during neuronal maturation, we utilized publicly available datasets (GSE151346) comprising single-cell RNA sequencing data of whole olfactory mucosa tissue dissected from mice (Brann et al., 2020). Our focus was on specific clusters containing the neuronal lineage, including GBCs, immediate neuronal precursors (INP), iOSNs, and mOSNs. By identifying clusters enriched with canonical cell markers, we isolated these clusters for all downstream analyses.

Normalization was performed to account for differences in sequencing depth, and highly variable genes were selected for further analysis. The data were log-transformed to stabilize variance across the dataset. Since the developmental time point at which ORs are properly folded and transported occurs during the development of OSN lineages, our focus was not to simply compare gene expression between cell types but rather along the continuum of neuronal maturation. Therefore, we employed DPT analysis (Wolf et al., 2018), which allowed us to order cells along a developmental trajectory, providing a linear representation of gene expression changes over neuronal maturation.

To facilitate the identification of genes that are either coregulated or antiregulated with any gene of interest across the neuronal developmental lineage or those enriched in specific cell types, we packaged our findings into an online application called geLine (https://github.com/Justice-Lu/geLine_dash). This interactive tool enables efficient parsing and detailed examination of specific gene expression profiles across the neuronal lineage using various count metrics.

### mOSN markers at different onset times and trajectory show different functions

To identify genes associated with alleviating ER stress and facilitating the cell surface expression of ORs, we aimed to characterize genes specifically enriched in mOSNs. Our analysis of the top 100 mOSN-enriched genes revealed distinct patterns in their activation and onset times along the maturation DPT trajectory (Fig. 1*B*). These genes were categorized into early- to late-onset groups based on their activation timing. Notably, gene activation timing across the maturation trajectory positively correlated with their overall enrichment in mOSNs (Fig. 1*D*).

To further understand the functional implications of these different trajectories, we performed Gene Ontology (GO) analysis on the top 100 mOSN-enriched genes. This analysis showed that GO term clusters are distinctly contributed to by genes depending on their trajectories (Fig. 1*C*). Specifically, genes with early-onset activation contribute to different GO terms compared with those with late-onset activation, suggesting inherent functional differences based on activation timing.

We further investigated the functional distinctions between early- and late-onset genes by cross-mapping with bulk-RNA-seq datasets differentially expressed for sorted mOSNs with high and low ER stress (Shayya et al., 2022). We found that early-onset mOSN genes are enriched in mOSNs exhibiting high ER stress, while late-onset mOSN markers are enriched in mOSNs with lower ER stress levels (Fig. 1*E*). Interestingly, genes with earlier onset are preferentially enriched in high ER stress mOSNs, coinciding with the trajectory of Rtp1.

Next, we asked which transcription factor(s) might be regulating genes that are coregulated during OSN lineage. Among the top 100 genes enriched in mOSNs, Nfe2l2 is the only known transcription factor. Utilizing the ARACNE-AP (Algorithm for the Reconstruction of Accurate Cellular Networks using Expression Profiling) to construct a gene regulatory network, we discovered that Nfe2l2 shows direct regulatory interactions with 64 out of the top 100 mOSN-enriched genes (Fig. 1*F*). ARACNE-AP identifies these interactions by employing mutual information metrics to eliminate indirect interactions, focusing only on direct regulatory links between a transcription factor and its target genes (Margolin et al., 2006). The discovery that Nfe2l2 is directly connected to majority of the mOSN markers underscores the efficacy of utilizing transcription factors and ARACNE-AP to identify functional related genes. This targeted approach, driven by ARACNE-AP, can be utilized more broadly to identify genes regulated by other phenotypes, such as ER stress transcription factors. By doing so, we can uncover additional regulatory networks and potential chaperones critical for various cellular processes, providing a comprehensive understanding of gene regulation in different contexts.

### Identification of coregulated genes with Rtp1 in OSN lineage

**Figure 2.**
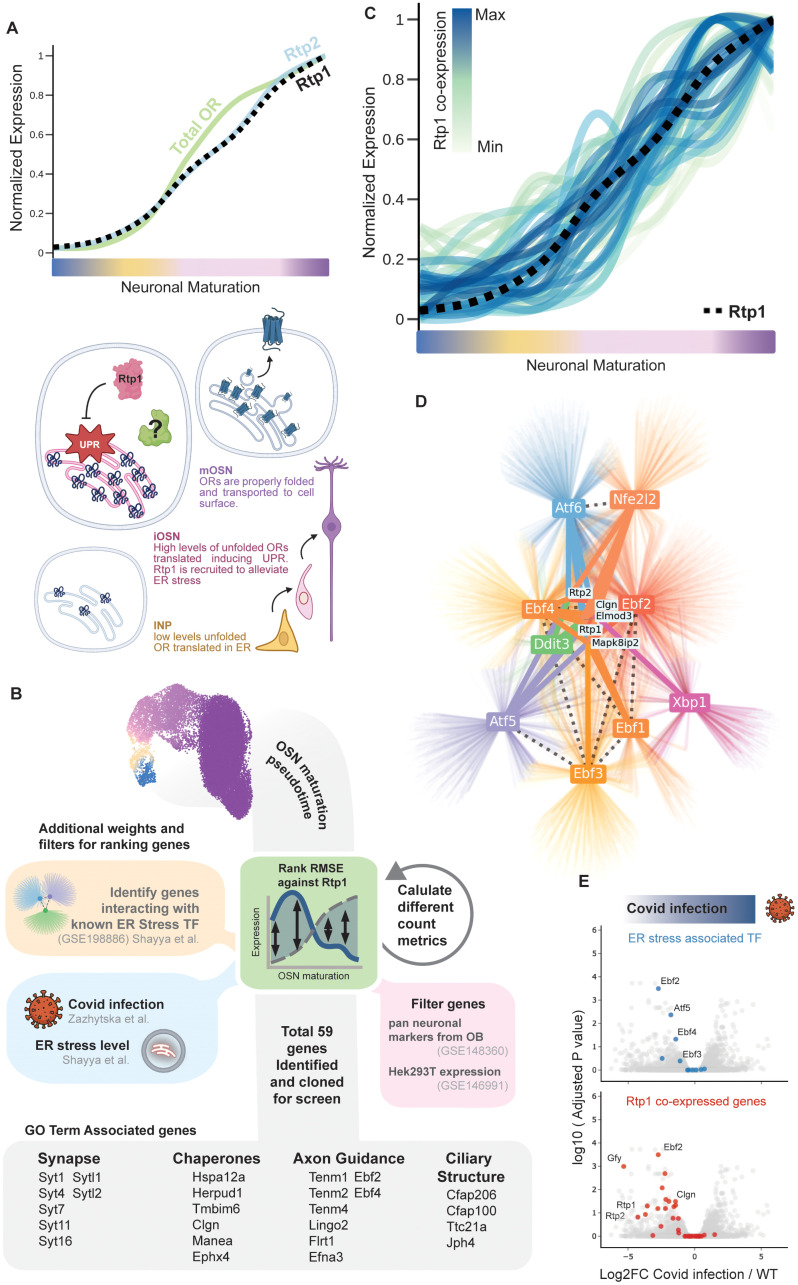
Identification of coregulated genes with Rtp1 in OSN lineage. ***A***, Schematic of olfactory sensory neuron (OSN) maturation stages, illustrating the unfolded protein response (UPR) during early differentiation. Rtp1 and hypothesized unidentified chaperones are recruited to alleviate ER stress, facilitating receptor trafficking and maturation. ***B***, Overview of the in silico pipeline used to identify genes coregulated with Rtp1. Single-cell RNA-seq data from mouse olfactory mucosa were used to quantify gene expression across OSN development. Additional datasets (ER stress ARACNE network, bulk RNA-seq under ER stress, mouse olfactory bulb RNA-seq, and bulk RNA-seq from COVID-19-infected human olfactory epithelium) were incorporated for filtering and weighting gene candidates. Final gene candidates are listed with their associated Gene Ontology (GO) terms. ***C***, Gene expression dynamics along the OSN lineage shown using geLine. The *x*-axis represents OSN maturation binned by DPT, while the *y*-axis shows normalized gene expression levels. Rtp1 expression is shown as a dotted black line. Other lines represent genes with similar expression dynamics, color-coded by correlation strength with Rtp1. ***D***, ARACNE-PE-derived gene regulatory network centered on ER stress-associated transcription factors (ER TFs). Colored nodes represent ER TFs, lines indicate gene–TF interactions, and bold lines highlight the top TF–gene interactions (white boxed nodes). Dotted lines show TF–TF regulatory connections. ***E***, Volcano plot of differential gene expression from bulk RNA-seq of olfactory epithelium autopsy samples from COVID-19 patients. Top, Volcano plot with red dots represent the top 50 Rtp1-associated genes, while bottom, blue dots highlight ER stress-associated transcription factors. With both category downregulated via COVID infection.

Given the previous findings that Rtp1 is highly expressed in mOSNs with high ER stress, presumably to alleviate this stress, and that Rtp1, along with the closely related Rtp2, shows nearly identical expression trajectories (Fig. 2*A*, Fig. S1), this consistent pattern suggests that additional elusive chaperones collaborating with Rtp1 might also follow a similar expression trajectory. Therefore, we aimed to trace the expression of Rtp1 in hopes of identifying additional molecular chaperones that also facilitate the cell surface expression of ORs in the heterologous cell system.

Following DPT analysis, pseudotime values of individual cells were mapped back to the raw count gene expression matrix to identify genes coregulated with Rtp1 during the maturation of the OSN lineage (Fig. 2*C*). We applied a variety of normalization metrics, including normalized counts, logmaritize counts, normalized counts adjusted for the maximum expression of each gene, and similarly adjusted logmaritize counts. These different normalization methods ensured that our analysis was not biased toward specific genes due to variations in expression counts (Fig. S2).

Subsequently, we filtered the coregulated gene candidates by excluding pan-neuronal genes identified from top genes enriched in neurons in the olfactory bulb scRNAseq dataset (GSE148360; Brann et al., 2020) to eliminate genes that naturally increase expression in nonolfactory neurons. The top 50 genes identified from the different metrics are represented on various axes to demonstrate their ranking across different metrics.

We then explored the relationship between genes coregulated with Rtp1 and their correlation with ER stress transcription factors. To achieve this, we utilized another publicly available dataset of the OSN transcriptional network (GSE198886; Shayya et al., 2022), which was inferred using ARACNE-AP. From the transcriptional network, we specifically investigated ER stress-associated transcription factors (Atf5, Atf6, Ddit3, Ebf1, Ebf2, Ebf3, Ebf4, Nfe2l2 and Xbp1; Shen and Prywes, 2005; Sammeta and McClintock, 2010; McClintock, 2015; Shayya et al., 2022). Consistent with our expectation, the analysis revealed that Rtp1 and Rtp2 were among the most interacted genes with the ER transcription factors (Fig. 2*D*), connecting with seven and six out of the nine ER stress transcription factors, respectively. More interestingly, we also identified Clgn, a homolog of the known ER chaperon calreticulin (Calr; Dey and Matsunami, 2011; Jiang et al., 2014; Tokuhiro et al., 2015), interacting with six of the transcription factors. This finding highlights the significant role of genes identified in the network in the context of ER stress, suggesting their potential involvement in ER stress and potential in increasing cell surface expression.

Previous studies have observed that during SARS-CoV-2 infection in the human OE, there is a reduction in the expression of mOSN markers (Zazhytska et al., 2021, 2022). Interestingly, not all mOSN markers are downregulated. The genes that are significantly downregulated include those important for OR signaling and trafficking, specifically Rtp1 and Gfy (Fig. 2*B*). Thus, we were interested in further examining other correlations of our identified genes of interest with SARS-CoV-2 infection. This comparison demonstrated a systematic downregulation of the ER stress-associated transcription factors and the top genes coregulated with Rtp1 in the context of SARS-CoV-2 infection. Among the top SARS-CoV2 infection downregulated genes that is also coregulated with Rtp1, we also identified Clgn again as one of the genes significantly downregulated from infected patients. This consistent downregulation provides further confidence in the potential of these genes and justifies pursuing further research to test their role in increasing cell surface expression in cell culture systems.

### Gfy and Clgn combination is important for enhanced cell surface expression of ORs

From the list of candidate genes identified in the previous analysis, we first identified genes with known associations to olfaction in the literature. Through this process, we identified Goofy (Gfy) and Calmegin (Clgn). As Gfy has been implicated in altering olfactory function, and disruption of Gfy results in abnormal localization of Adenylyl cyclase type 3 (Adcy3), shortened olfactory cilia development and reduced sensitivity to odorants (Kaneko-Goto et al., 2013). Clgn, in addition to being a homolog of Carl, has also been identified as a chaperone required for fertile spermatogenesis and shows robust expression in OSN (Dalton, 2017). However, neither Gfy nor Clgn has been previously implicated or demonstrated to facilitate the cell surface expression of ORs.

**Figure 3.**
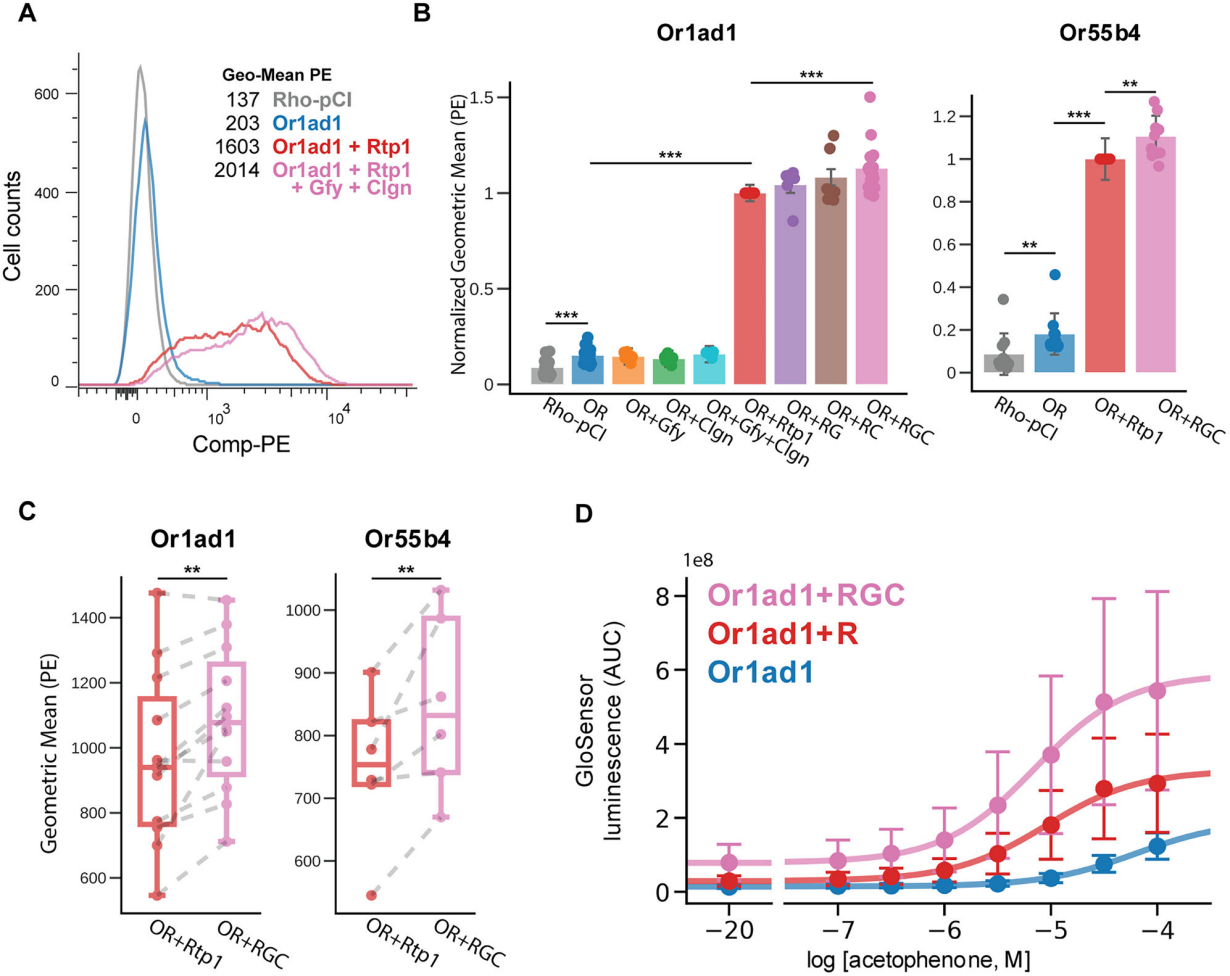
Gfy and Clgn cooperate with Rtp1 to enhance OR surface expression. ***A***, Flow cytometry analysis of Or1ad1 cell surface expression in heterologous cells. Expression is low with OR alone and increased with cotransfection of Rtp1, further enhanced with the combination of Rtp1, Gfy, and Clgn. Rho-pCI empty vector served as negative control. Phycoerythrin (PE) fluorescence was used to evaluate surface expression, and geometric mean (Geo-Mean) PE intensity is shown. ***B***, Quantification of OR surface expression based on PE fluorescence intensity under various transfection conditions for Or1ad1 (left) and Or55b4 (right). Statistical significance was assessed using the Wilcoxon rank-sum test (scipy.stats.ranksums). ***C***, Paired comparison of Geo-Mean PE intensity between two experimental conditions on the same date (linked by gray dotted lines). Statistical significance was evaluated using a paired *t* test (scipy.stats.ttest_rel). Data are presented as box plots with median, interquartile range, and individual data points. ***D***, Real-time Glosensor cAMP assay monitoring Or1ad1 activation by acetophenone across different accessory protein cotransfections. Abbreviations: OR, olfactory receptor; RG, Rtp1s + Gfy; RC, Rtp1s + Clgn; RGC, Rtp1s + Gfy + Clgn. Data are represented as mean ± SE, *p < 0.05, **p < 0.01, ***p < 0.001.

To evaluate the effect of these genes on the cell surface expression of specific ORs, we utilized flow cytometry to quantify the cell surface expression of ORs transiently expressed in HEK293T cells. Our initial experiments demonstrated that neither Gfy nor Clgn alone was able to increase cell surface expression of the ORs (Fig. 3*B*). Additionally, even when expressing Gfy or Clgn individually in combination with Rtp1, we did not observe an enhancement in cell surface expression. However, when both Gfy and Clgn were coexpressed with Rtp1, we observed a modest but consistent increase in cell surface expression compared with the expression of Rtp1 alone (Fig. 3*A*). This increase is observed consistently across multiple experiments.

We further replicated the experiment using two different types of receptors, Or1ad1 (also known as Olfr1377 and MOR129-1) and Or55b4 (also known as OR-S6, Olfr544 and MOR42-3). In both cases, the coexpression of Gfy and Clgn with Rtp1 resulted in a similar increase in cell surface expression (Fig. 3*C*). These findings suggest that while Gfy and Clgn alone or individually combined with Rtp1 do not enhance OR surface expression, their combined presence with Rtp1 leads to a modest yet consistent increase in surface expression.

### Enhanced cell surface expression of ORs by synaptotagmins

**Figure 4.**
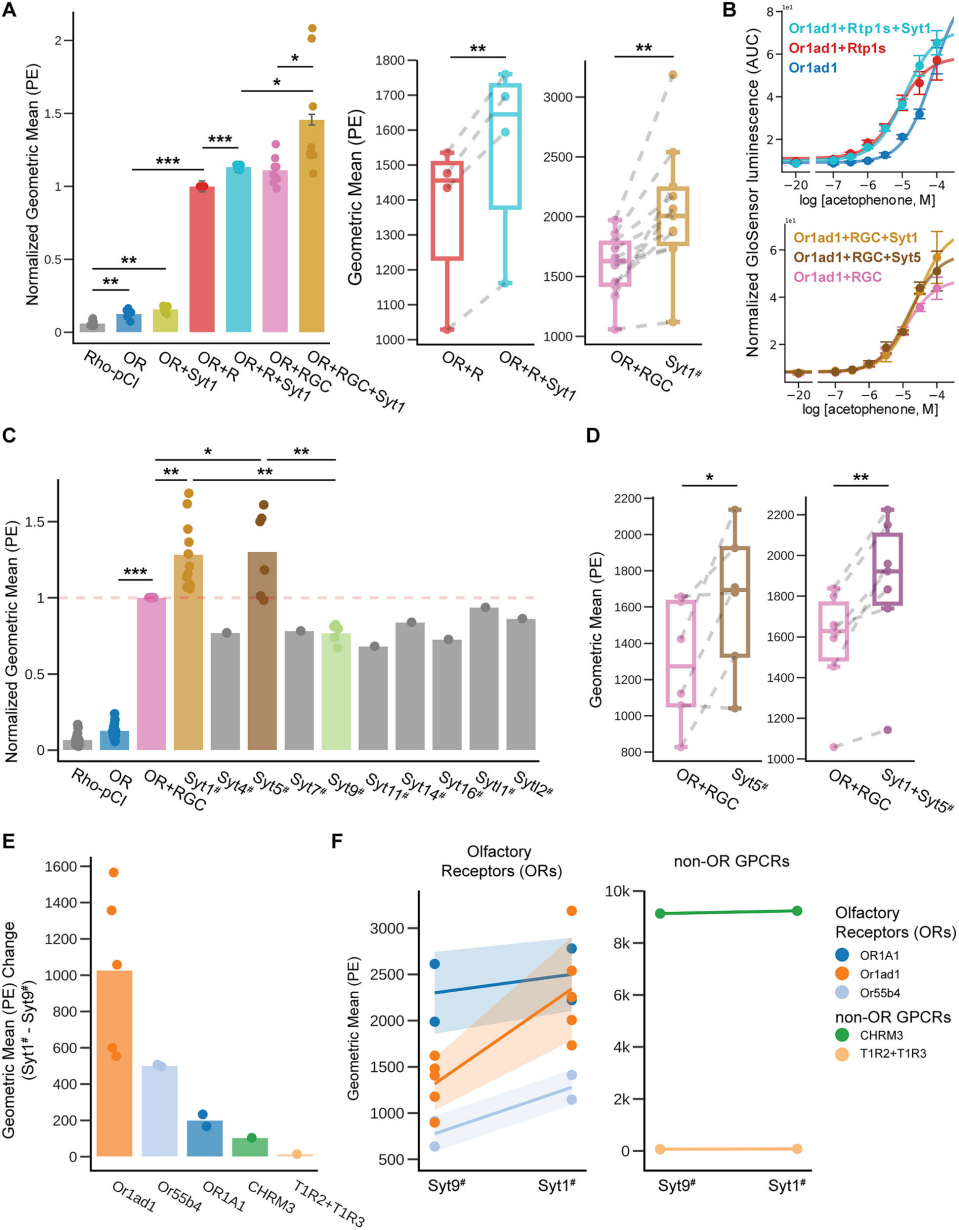
Synaptotagmins enhance Or1ad1 cell surface expression. ***A***, Left, Quantification of Or1ad1 surface expression based on PE fluorescence by flow cytometry. Right, Paired comparison of Geo-Mean PE intensity on matched experimental dates (gray dotted lines). RG, RC, and RGC combinations are as defined in Figure 3. ***B***, Glosensor cAMP assay showing real-time response of Or1ad1 to acetophenone with different accessory proteins. ***C***, Normalized PE intensity relative to OR + RGC condition (indicated by red dashed line). ***D***, Box plot comparing Geo-Mean PE levels between paired conditions on the same date. Gray dotted lines link matched experimental runs. ***E***, Bar plot comparing surface expression of difference between ORs and non-OR GPCRs cotransfected with either Syt1 or Syt9. ***F***, Geo-Mean PE levels for individual ORs (Left) and non-OR GPCRs (Right) across Syt1 and Syt9 conditions in paired experiments. Hashtag (#) denotes inclusion of OR + RGC. Abbreviations: OR, olfactory receptor; RGC, Rtp1s + Gfy + Clgn. Data are represented as mean ± SE, *p < 0.05, **p < 0.01, ***p < 0.001.

Among the genes coregulated with Rtp1, we identified Synaptotagmin 1 (Syt1) as another important modulator of OR surface expression. While Syt1 is a well-known calcium sensor involved in neurotransmitter release and synaptic vesicle exocytosis (Bacaj et al., 2013; Li et al., 2017; Volynski and Krishnakumar, 2018), its function in OSNs had not been previously documented. Our investigation revealed that transient coexpression of Syt1 with Rtp1 in HEK293T cells significant increase in cell surface expression of Or1ad1 (Olfr1377; Fig. 4*A*). Building on this finding, we further tested whether the increase in surface expression could be enhanced by additional accessory factors. Indeed, coexpression of RGC together with Syt1 led to even greater enhancement of Or1ad1.

We expanded our investigation to other synaptotagmin family members with detectable expression in OSN lineage, including Syt1, Syt4, Syt5, Syt7, Syt9, Syt11, Syt14, Syt16, Sytl1, and Sytl2. Most did not enhance OR surface expression when coexpressed with Rtp1, Gfy, and Clgn (RGC), except Syt1 and Syt5, both of which significantly increased the surface expression of Or1ad1 (Fig. 4*C*,*D*). Although less well characterized, Syt5 has also been implicated in calcium-dependent neuronal exocytosis (Lenzi et al., 2019), supporting the idea that certain synaptotagmins may have specialized roles in OR membrane trafficking.

To test whether Syt1 affects OR function, we compared acetophenone dose–response curves of Or1ad1 expressed with Rtp1 alone versus with Rtp1 and Syt1. The addition of Syt1 only minimally increased the maximal activation at high ligand concentrations, it did not affect the EC50 (Fig. 4*B*), suggesting that Syt1 enhances surface trafficking or receptor abundance, rather than altering receptor sensitivity or intrinsic activity.

To further evaluate the specificity of Syt1's effect, we compared Syt1 with Syt9 as a negative control. Syt9 was selected because it is expressed in neither neurons nor HEK293T cells (Fig. S3*B*,*C*). Using a panel of receptors (OR1A1, Or1ad1, and Or55b4) with various expression levels, as well as two nonolfactory GPCRs (taste receptor T1R1 + T1R3 and acetylcholine receptor CHRM3), we assessed cell surface expression when coexpressed with Rtp1, Gfy, and Clgn (RGC), alongside either Syt1 or Syt9. The result showed that the Syt1's effect varied by receptors, while consistently enhancing surface expression for ORs (Fig. 4*E*,*F*). Importantly, Syt1 did not enhance the surface expression of the tested non-OR GPCRs. Together, these results indicate that Syt1's effect is not due to a general perturbation of ER/Golgi function or nonspecific enhancement but instead reflects a receptor-specific role in OR trafficking.

**Figure 5.**
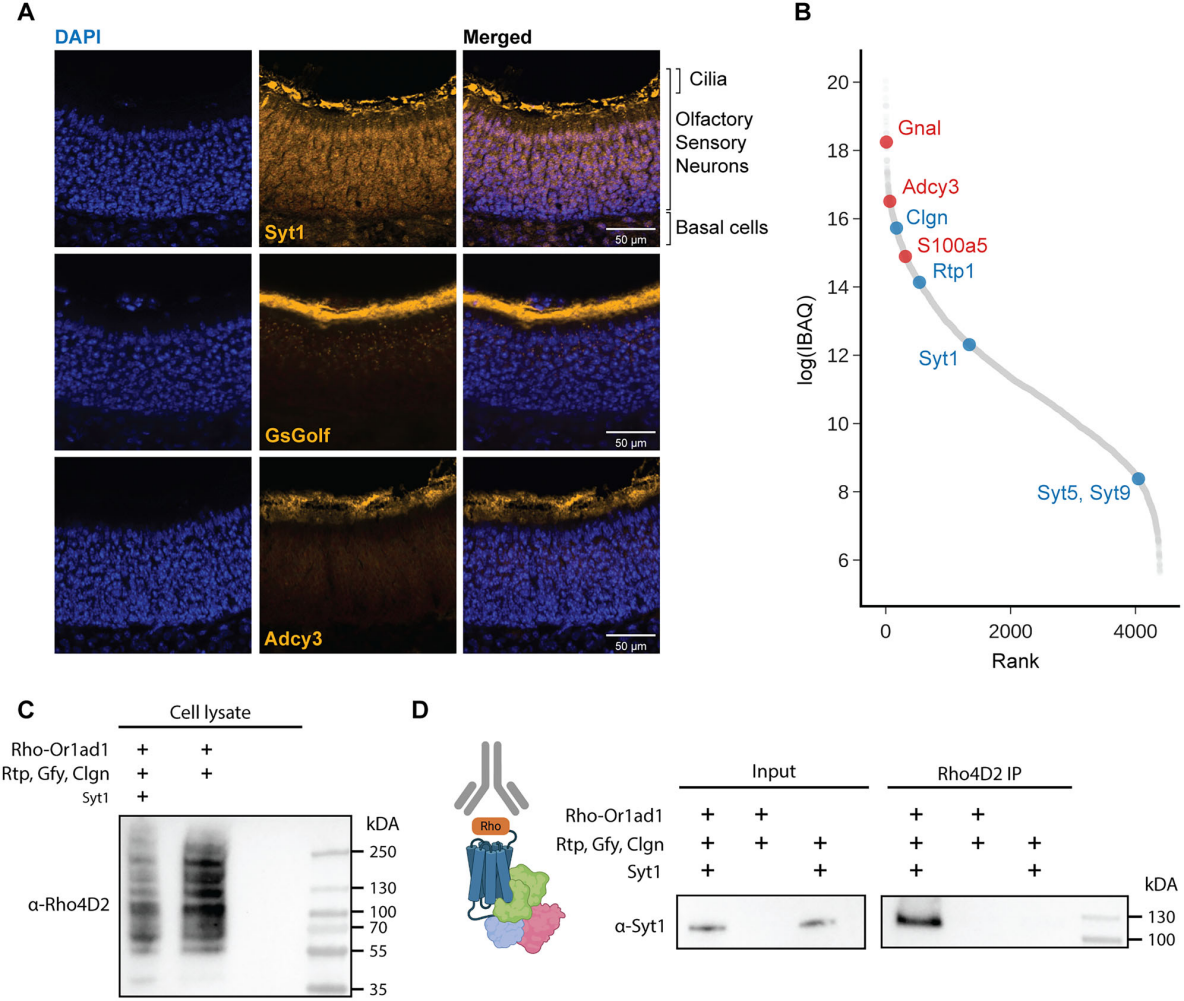
Syt1 shows expression in Cilia of OSNs and interacts with Or1ad1. ***A***, Immunohistochemical staining of mouse olfactory epithelium shows Syt1 colocalization with canonical olfactory cilia markers Gnal (GsGolf) and Adcy3, suggesting its presence in the cilia of olfactory sensory neurons (OSNs). ***B***, Proteomic analysis of isolated olfactory ciliary membrane fractions reveals proteins ranked by descending mean iBAQ values. Canonical olfactory cilia membrane proteins are highlighted in red, and proteins identified in this study are highlighted in blue. ***C***, Western blot confirms presence of Or1ad1 in both OR + RGC (middle lane) and OR + RGC + Syt1(left most lane) transfection HEK293T-cell lysates contain Or1ad1, but not in no transfection control (right most lane). With cotransfection of Syt1 decreasing overall Or1ad1 protein expression comparably. ***D***, Left, Input lysate Western blot confirms equivalent Syt1 protein levels across samples. Right, Co-immunoprecipitation (co-IP) with Rho4D2 antibody shows a specific interaction between Syt1 and Or1ad1. Negative controls lacking either the OR or Syt1 show no detectable interaction.

To assess whether Syt1 alters OR protein levels, we conducted Western blot analysis of Or1ad1 coexpressed with RGC alone or RGC plus Syt1, using an antibody against the N-terminal Rho-tag for the OR. Syt1 coexpression did not increase total Or1ad1 protein levels (Fig. 5*C*), consistent with the interpretation that the observed increase in cell-surface expression reflects more efficient trafficking and/or folding rather than elevated OR production.

We next asked whether Syt1 physically associates with ORs. We performed co-immunoprecipitation (co-IP) experiments using Rho-tagged Or1ad1 and anti-Rho antibody for pulldown revealed a robust Syt1 signal in the co-IP fraction when Or1ad1 was coexpressed with RGC and Syt1 (Fig. 5*D*). Controls lacking either Or1ad1(RGC + Syt1) or Syt1 (OR + RGC) did not yield detectable Syt1 in the pull down, despite comparable Syt1 input levels in the lysates (Fig. 5*D*), supporting a specific interaction, either direct of complex-mediated, between Syt1 and Or1ad1.

Finally, immunohistochemistry (IHC) on mouse olfactory epithelium revealed robust expression of Syt1 in the OSN cilia layer, a site for odor detection and OR localization (Fig. 5*A*). Analysis of a published olfactory cilia proteome dataset (Kuhlmann et al., 2014) further confirmed Syt1 enrichment, alongside canonical olfactory signaling proteins such as G alpha olf (Gnal) and Anenylyl cyclase type 3 (Adcy3; Fig. 5*B*).

Taken together, our results identify Syt1 as a novel modulator of OR surface trafficking. Beyond its canonical role in synaptic vesicle release, Syt1 facilitates OR expression at the plasma membrane in the presence of other accessory proteins, suggesting a previously unrecognized role for Syt1 in the trafficking of ORs in OSNs.

## Discussion

Continued efforts to elucidate the molecular mechanisms underlying OSN maturation have been significantly advanced by leveraging single-cell RNA sequencing and computational tools. Despite substantial progress, the functional expression of ORs and the identification of accessory molecules that enhance OR surface expression remain challenging. To address these issues, we employed DPT analysis and developed the geLine tool to investigate gene expression trajectories of OSNs. This approach enabled the identification of key regulatory genes, including those coregulated with the receptor transporting protein 1 (Rtp1), and allowed assessment of their potential roles in facilitating OR surface expression. Our study demonstrates the utility of integrating publicly available datasets with computational approaches to uncover associative genes, such as molecular chaperones like Rtp1, which can be further validated in heterologous expression systems.

We discovered a positive correlation between gene onset timing and their enrichment in mOSNs (Fig. 1*D*). Moreover, mOSNs exhibiting high ER stress showed upregulation of early-onset genes, while later-onset genes were comparatively downregulated (Fig. 1*E*). This underscores the complex interplay between temporal gene expression and cellular stress responses during OSN maturation. Notably, the molecular chaperones Rtp1 and Rtp2 (Saito et al., 2004), previously shown to facilitate OR surface expression, are among the early-onset genes enriched in high ER stress mOSNs. These observations suggest that additional molecular chaperones functioning alongside Rtp1 may follow similar expression trajectories, reflecting a coordinated response to ER stress during OR maturation.

Functional studies identified Goofy (Gfy) and Calmegin (Clgn) as promising accessory proteins that enhance OR surface expression. Gfy is implicated in olfactory function, with knock-out studies showing reduced odor detection thresholds in mice (Kaneko-Goto et al., 2013). Clgn, a homolog of the ER chaperone calnexin, is involved in protein folding and quality control, particularly in spermatogenesis (Lu et al., 2003; Tokuhiro et al., 2015). Although neither gene alone nor in combination with Rtp1 significantly increased OR surface expression, coexpression of Gfy and Clgn with Rtp1 produced a modest but consistent enhancement. This suggests a synergistic interaction, where these proteins together facilitate trafficking and stabilization of ORs at the cell surface. The dependence of this effect on the combined presence of these chaperones highlights the importance of multiprotein coordination for optimal OR maturation and expression.

### Role of synaptotagmin in OSN

Unexpectedly, we identified Synaptotagmin 1 (Syt1) as a modulator of OR surface expression. The synaptotagmin (Syt) family comprises multiple isoforms involved in calcium-dependent membrane trafficking (Li et al., 2017; Volynski and Krishnakumar, 2018; Lenzi et al., 2019). Each isoform mediates vesicle fusion and membrane interactions with distinct functional and spatial roles. For example, Syt1 functions as a low-affinity Ca^2+^ sensor, primarily involved in fast, synchronous neurotransmitter release, while Syt7 mediates asynchronous release triggered by persistent Ca^2+^ elevation.

Our single-cell RNA-seq analysis revealed that Syt1 is highly expressed in OSNs and tightly coordinated with Rtp1 (Fig. S3*A*, Table 2), unlike other Syt members that show broader expression across non-neuronal cell types and lack synchrony with Rtp1 (Fig. S3*B*).

**Table 2.** Rtp1 coexpressed genes cloned

	log1p counts	Normalized log1p counts	Raw counts	Normalized counts
Acsl6	0.02574891	0.00635007	0.84167958	0.00461251
Aplp2	1.10476097	0.01261855	79.6242884	0.00343709
Brdt	0.1280126	0.01709209	3.13860248	0.02141941
Ccdc184	0.2070298	0.05454451	4.94316878	0.09705289
Cfap100	0.31223916	0.05080132	6.34602559	0.03078239
Cfap206	0.15334059	0.00378982	3.57796221	0.00346708
Chga	1.01494222	0.03361486	155.520835	0.05542413
Chgb	0.31118729	0.01637929	6.42438727	0.0078254
Clgn	0.74091267	0.00318404	56.6399861	0.00657914
Clstn2	0.09337924	0.00416458	2.33459514	0.00361567
Ddit3	0.14625503	0.0457801	2.62840796	0.03517463
Ebf2	1.98567918	0.19901103	110.019129	0.22353363
Ebf4	0.14691291	0.03405947	3.76809551	0.04623054
Efcab10	0.13477889	0.00266032	3.3859207	0.00215273
Efna3	0.28493066	0.09458243	6.04480429	0.08326113
Ephx4	0.15409435	0.04314291	2.9406858	0.04010401
Esyt2	0.50511688	0.2152819	9.83442767	0.21202229
Faim2	0.06421857	0.04934433	1.05442355	0.03764771
Fam217a	0.10150038	0.01015655	2.10829769	0.00483947
Flrt1	0.55172245	0.02355901	32.1677279	0.03715464
Gfy	0.99665555	0.00489499	80.5252713	0.00330078
Gpx6	0.60875522	0.39881186	11.2951948	0.37295985
Herpud1	0.13226391	0.14463243	1.99241105	0.10349006
Hspa12a	0.50381504	0.00789698	9.82695138	0.00696805
Jakmip1	0.03805607	0.02846886	0.74569328	0.02096921
Jph4	0.570976	0.20369152	10.8409687	0.22459363
Klhl5	0.01353289	0.00851615	0.2983265	0.00917816
Lingo2	0.24420541	0.07854429	5.37514231	0.09156097
Manea	0.0410667	0.02782741	0.58235434	0.020182
Pde6d	0.02411912	0.01014788	0.41065243	0.00598411
Rd3	0.61315523	0.13672843	11.3490718	0.11931109
Rtbdn	0.60334628	0.31213814	11.2147253	0.26529135
Serpine2	0.84411171	0.01824223	115.428822	0.02381207
Slc24a2	0.10863693	0.00547784	2.17211587	0.00498321
Snap25	0.00953043	0.00170627	0.31162994	0.00174173
Stx1a	0.32236121	0.21968956	7.18081421	0.24276433
Stx1b	0.59716298	0.31174404	11.168392	0.32196071
Sult1d1	0.57425573	0.00533355	58.4882083	0.00309386
Syt1	0.00824814	0.00929026	0.17725211	0.00716922
Syt11	1.0668535	0.07460285	53.8653001	0.0829906
Syt14	0.42069153	0.1475637	8.51866478	0.16142159
Syt16	0.51232898	0.07625909	9.92989837	0.09304215
Syt4	0.18874748	0.01126145	3.83015945	0.01782046
Syt5	0.30340706	0.18640631	6.89704877	0.20644688
Syt7	0.07775506	0.03558224	2.10025604	0.04306901
Syt8	0.61542539	0.47208121	11.3974767	0.42137981
Syt9	0.60186781	0.39674091	11.2095174	0.33785704
Sytl1	0.39554346	0.04555998	8.14676721	0.07397757
Sytl2	0.24915932	0.04932683	4.86608161	0.0405874
Tenm1	0.57307727	0.11037101	10.8524228	0.1245037
Tenm2	0.12478065	0.06240682	8.16974792	0.09047471
Tenm4	0.36799326	0.245549	7.48509798	0.26240721
Tmbim6	0.71797144	0.02511061	63.3963663	0.009451
Ttc21a	0.12457681	0.00624712	2.91953405	0.00392327
Umodl1	0.16405443	0.11516587	2.60110127	0.08161242
Vamp1	0.47403176	0.02989891	9.37587177	0.03517849
Vamp2	0.17423627	0.06703294	4.63343734	0.09306683
Syt6	0.61619634	0.55155568	11.4034411	0.42471957
Rtp1	0	0	0	0

This table lists all genes cloned and functionally tested in this study, alongside their root mean square error (RMSE) values calculated against Rtp1 expression trajectory. RMSE values are reported across different normalization methods: log-transformed counts (log1p), log1p with max expression normalization, raw counts, and counts with max expression normalization. These metrics reflect the degree of expression similarity to Rtp1 across OSN maturation.

Functionally, coexpression of Syt1 in heterologous cells significantly increased the surface presentation of Or1ad1, but only in the presence of Rtp1 (Fig. 4*A*–*D*), suggesting a cooperative role between Syt1 and Rtp1 in membrane delivery. Among the Syt family members tested, only Syt5 showed a similar capacity to enhance OR surface expression (Fig. 4*C*), underscoring the functional specificity of Syt1 within the OSN context. Extending this analysis, we observed that while the effect of Syt1 varied across different ORs, it did not enhance surface expression of the non-OR GPCRs tested (Fig. 4*E*,*F*), indicating receptor-selective rather than global ER/Golgi effects. Importantly, once an OR reaches sufficient levels at the cell surface, further increases in expression may not yield proportional gains in receptor activation as shown in Or1ad1 (Fig. 4*B*), reflecting a plateau effect due to receptor saturation. However, for poorly expressed ORs, even modest improvements in trafficking or surface localization can be critical. In such cases, Syt1 may uncover measurable functional responses where none would otherwise be detected, making its contribution particularly relevant for ORs with low intrinsic surface expression.

Additionally, we confirmed that HEK293T cells do not endogenously express detectable levels of Rtp1, and they express low expression Gfy, Clgn, and Syt1 (Table 3, Fig. S3*C*). Therefore, the modest increase in OR surface observed in the heterologous system may be attributable to the already low levels of endogenous expression of these accessory proteins. Proteomic analysis of purified OSN cilia (Kuhlmann et al., 2014) revealed robust Syt1 enrichment alongside canonical signaling components Gnal, Adcy3, and S100a5, corroborated by IHC (Fig. 5*A*,*B*). This ciliary localization suggests that Syt1 may facilitate OR trafficking and localization at sensory cilia, extending its known synaptic vesicle function to OR maturation.

**Table 3.** Summary of TPM expression in Hek293T cells

	gene_symbol	TPM
ENSG00000111640_GAPDH	GAPDH	2,072.09
ENSG00000075624_ACTB	ACTB	1,690.6
ENSG00000175077_RTP1	RTP1	0
ENSG00000153132_CLGN	CLGN	8.205
ENSG00000127022_CANX	CANX	497.17
ENSG00000179218_CALR	CALR	549.97
ENSG00000261949_GFY	GFY	0.315
ENSG00000067715_SYT1	SYT1	0.225
ENSG00000129990_SYT5	SYT5	0.48
ENSG00000170743_SYT9	SYT9	8.86

Transcript per million (TPM) counts of selected chaperone genes extracted from the GSE132044 bulk dataset, limited to HEK293 cells. The listed genes correspond to those tested in this study and include canonical housekeeping controls (GAPDH, ACTB), Clgn homologs (CANX, CALR), and additional candidate chaperones (Rtp1, Gfy, Clgn, Syt1, Syt5, Syt9). These values provide the underlying expression data for Figure S3*C*.

Co-immunoprecipitation revealed Syt1 associates with ORs only when coexpressed with ORs and RGC (Or1ad1 + RGC + Syt1; Fig. 5*D*). Controls lacking OR or Syt1 showed no signal. Interestingly, Syt1 also co-immunoprecipitated with CHRM3, despite not enhancing its surface expression in our flow cytometry assays (Fig. S4*C*). This discrepancy may reflect that CHRM3, the highest expressed receptor in our study, is already expressed near saturation. A notable observation in our co-IP results was a shift in the apparent molecular weight of Syt1: the co-IP fraction was enriched for higher molecular weight variants compared with the input lysate (Fig. S4*C*). Though unexpected, the shift may reflect multimeric forms of Syt1, which are known to form stable oligomeric structures under conditions of calcium binding and membrane association (Wang et al., 2014; Wolfes and Dean, 2020). Such multimeric forms could selectively participate in complex formation with ORs, providing an explanation for the observed shift.

Overall, our findings uncover a novel, noncanonical role for Syt1 in the maturation and trafficking of ORs. While Syt1 has been extensively studied in synaptic vesicle dynamics (Bacaj et al., 2013; Wang et al., 2014; Li et al., 2017; Volynski and Krishnakumar, 2018; Lenzi et al., 2019; Wolfes and Dean, 2020), our results indicate that its function extends membrane protein trafficking in OSNs. This context-specific physical interaction with ORs, along with its synergy with known OR accessory proteins, suggests that synaptotagmins, particularly Syt1, may serve as previously unrecognized components of the OR maturation, broadening our understanding of the molecular mechanisms underlying OR expression.

### Functions for accessory proteins (Gfy, Clgn, and Syt1) for OR surface expression

ORs undergo complex ER folding, quality control, and trafficking processes, often triggering the UPR to ensure proper conformation (Shen and Prywes, 2005; Sammeta and McClintock, 2010; Shayya et al., 2022). Rtp1 facilitates OR transport from the ER to the plasma membrane, contributing to both trafficking and transcriptional regulation for OR gene choice (Ikegami et al., 2020; Zazhytska et al., 2022).

Tracing genes coregulated with Rtp1 during OSN maturation revealed Gfy, Clgn, and Syt1 as accessory proteins that enhance OR surface expression when combined with Rtp1 (Fig. 4*C*–*E*). Gfy localizes to the OSN Golgi and supports Adcy3 positioning and olfactory cilia development (Kaneko-Goto et al., 2013). Clgn, a calnexin homolog, facilitates proper folding and presentation of client proteins (Dalton, 2017). Syt1 localizes to OSN cilia and likely supports OR trafficking beyond its classical synaptic role.

We propose a model in which Rtp1 acts as the primary ER chaperone, Gfy and Clgn enhance folding and stabilization during ER-to-Golgi transport, and Syt1 facilitates final trafficking to the plasma membrane. The coordinated action of these proteins increases OR surface expression efficiency. While the observed enhancements are modest, they are reproducible and physiologically relevant, particularly for poorly expressed ORs, and may reveal functional receptor activity otherwise undetectable.

### geLine Dash tool for investigating neuronal lineage expression trajectories

The geLine Dash tool integrates DPT (Wolf et al., 2018) with interactive mapping of gene expression across OSN maturation, enabling high-resolution visualization of dynamic gene trajectories beyond conventional cell-type binning. It is especially useful for identifying genes coexpressed with any gene of interest, facilitating exploration of potential regulatory networks and functional interactions (Fig. S1). geLine’s versatility allows application across multiple neuronal lineages, offering a powerful resource for studying differentiation, coregulation, and developmental timing of key genes.

### Implications and future directions

Our study highlights accessory proteins that enhance OR surface expression but notes that requirements may vary among ORs. Future work should characterize interactions between candidate genes and their roles in OR trafficking and stabilization across diverse receptor types. The ability to screen gene interactions in heterologous systems provides a platform for investigating OR–ligand interactions and identifying additional modulators of OR maturation.

In conclusion, by combining Rtp1 with Gfy, Clgn, and Syt1, we provide a framework to enhance OR surface expression and uncover functional roles for accessory proteins. Syt1 emerges as a novel, context-specific facilitator of OR trafficking, illustrating the power of integrating single-cell transcriptomics, computational tools, and functional assays to advance our understanding of OSN biology.
